# A child mental health service transformation framework in the Global South

**DOI:** 10.1177/13591045251366711

**Published:** 2025-08-19

**Authors:** Michelle O’Reilly, Panos Vostanis

**Affiliations:** 1 4488 University of Leicester, UK

**Keywords:** Children, youth, mental health, psychosocial, interventions, services, Global South

## Abstract

Global South countries are typically faced with multiple socioeconomic and resource challenges that predispose rising child mental health needs, which remain largely unmet. Collectivist societies, however, provide protective mechanisms for child mental health and opportunities for interventions through existing informal support systems. We provide an empirically grounded framework for child mental health service transformation, accounting for the complexities of resource burdens and the need for culturally sensitive adaptations. In presenting this framework, we utilise data from projects in South Africa and Pakistan which engaged participants from disadvantaged urban areas. These involved a total of 10 psychosocial interventions, 504 end-users (youth, parents and professionals) and a sub-sample of 76 focus group participants, to describe the design, refinement, cascade training, implementation and process evaluation of the framework. The secondary thematic analysis illustrates four layers of knowledge generation, translation and transfer, transformation through community engagement and mobilisation, and impact through service integration and systemic changes. The framework and supporting findings informed a provisional Theory of Change. This highlights the principles of stigma prevention, co-production with communities, contextualisation of psychosocial interventions, integration with informal and structural support systems, knowledge cascade, and involvement of youth and parents with lived experience.

## Plain language summary

Children and youth living in low- and middle-income countries, especially in areas of socioeconomic hardship, are faced with multiple environmental challenges that make them vulnerable to developing mental health needs. These needs are often not met, because of barriers in seeking help, and limited staff and resources. Collectivist societies, nevertheless, have strengths, such as informal support networks, that provide opportunities for the improvement of child mental health support. In this paper, we describe a framework through its different stages of design, refinement with local stakeholders, training, application, and evaluation of mechanisms that acted as barriers or enablers. We revisited data from projects in South Africa and Pakistan disadvantaged areas. In total, these involved 10 interventions and 504 beneficiaries (youth, parents and professionals), of whom 76 also participated in focus group discussions. Participants’ perspectives identified how child mental health knowledge and skills should be co-produced, translated to local needs and priorities, applied by involving communities, and integrating to existing support systems. This service model is guided by key principles of tackling stigma, communities owing solutions, combining psychological interventions with social support, and upskilling different provider groups, including youth and parents to act as links between communities and services.

## Introduction

Despite the increasing global policy attention to improving children’s mental health and wellbeing, countries in the Global South (also referred to as Low- and Middle-Income) are faced with multiple challenges in improving child mental health service provision (World Health Organisation, 2020). In terms of mental health needs, children are often exposed to additional risk factors than in western societies, especially those living in resource-constrained settings such as informal settlements. These compounding risk factors include a lack of basic needs such as sanitation and poor housing, unsafe neighbourhoods, recurrent exposure to violence within the family and community (polyvictimisation), political or ethnic conflict, competing economic pressures resulting in lack of parent engagement, adopting caregiver roles, and child labour (Honda et al., 2023; Le et al., 2018). Evidence suggests that females are likely to experience additional vulnerabilities of child marriage, sexual exploitation and lack of socio-educational opportunities for growth (Rumble et al., 2024). Stigma of mental health and mental illness, and underlying cultural concepts and beliefs, are common barriers to help-seeking (Khalil et al., 2020).

For those that do actively seek support for their mental health needs, and require service provision, there are challenges in the structures and systems. In terms of resources, those challenges are multidimensional. Interventions may not be culturally sensitive (Zhou et al., 2020) or lack a conceptual framework and/or evidence-base, especially for community implementation (Babatunde et al., 2021). Mental health infrastructure is constrained by variable policy and supporting budgets, staff numbers, professional roles and skills, adult-centric approaches, costs, and geographical barriers (Docrat et al., 2019).

Nevertheless, collectivist Global South societies also offer protective informal support mechanisms (Kohrt et al., 2018). Schools, early child development and youth centres, community- and religious-based organisations, and community volunteers or paraprofessionals can contribute to both preventive and responsive functions (Patel et al., 2018). Consequently, theoretically driven and community- or school-based psychosocial programmes have been developed to concurrently address awareness, welfare and mental health needs, with promising outcomes in building children’s and parents’ resilience and coping (Loades et al., 2024; Pedersen et al., 2019). For example, community involvement in co-production has shown to enhance engagement and retention (Nhedzi et al., 2022). Various Train-of-Trainer (ToT) training programmes have also been designed to cascade knowledge and skills to community and primary care groups within their existing roles, often combined with life skills education and health promotion (Leventhal et al., 2022).

This evidence highlights the importance of contextualised service frameworks that take into consideration barriers and enablers in Global South resource-constrained settings. To this effect, international policy increasingly adopts principles of stepped care, integration with current psychosocial support systems to maximise capacity, interdisciplinary joined up working, operating across the preventive-responsive spectrum, upskilling frontline professionals, involving communities in co-production and delivery, and endorsing digital approaches (ToC) (Galagali & Brooks, 2020; Kruk et al., 2022; World Health Organisation, 2014). However, such frameworks of integrated child mental health care have not, yet, been tested and evaluated in Global South resource-constrained settings. This paper describes the co-production, implementation and process evaluation of such a service transformation framework in two Global South Countries, South Africa and Pakistan, and presents the findings from the secondary analysis of two integrated datasets.

## Methods

The aim was addressed by the attending to the following research questions: (a) How do end-users, across two Global South resource-constrained settings, perceive and define mental health service transformation? (b) How do these local participants position themselves as holders and sharers of child mental health knowledge? (c) Which factors enable knowledge transfer to communities? (d) How could the findings inform a provisional Theory-of-Change?

Through this process, our goal was to develop a framework for child mental health service transformation, which is translatable and adaptable to other Global South resource-constrained contexts.

### Context and settings

The data are based on two studies that evaluated the process of implementation of a child mental health service transformation framework, which integrated mental health into existing psychosocial support through the Train-of-Trainer (ToT) approach, in two Global South countries, South Africa and Pakistan. This framework included a model of working, a training programme, and co-designed interventions founded in the framework and methodology (summarised below), and which are reported elsewhere (Vostanis et al., 2024a, 2024b). In this paper we illustrate, through a pedagogical narrative and empirical data, the evolution of the service transformation framework. We integrate two datasets from those inter-related projects, to address new research questions regarding the value of child mental health service transformation in the Global South (as above). The original research projects generating data had in place appropriate ethical governance and approvals, and secured consent from all necessary parties. 

Although this data synthesis was not framed as a comparative research design, the inclusion of two Global South sites could enable the identification of emerging cross-cutting or context-specific themes to inform service planning in other settings. Similarities between South Africa and Pakistan include high inequalities, poverty and associated risk factors, an increasing population of children, informal settlements, and inadequate service delivery (Organization for Economic Co-operation and Development, 2016). Potential differences include culture, nature of community supports, service systems, and traditional sources of help (Pakistan Bureau of Statistics, 2018; South African Child Gauge, 2024).

In South Africa, data were collected in the city of Ekurhuleni (East of Johannesburg) in Gauteng, with a population of 3,774,638, including five of the most 20 populated townships in the country (COGTA, 2020). In Pakistan, four disadvantaged areas in the city of Karachi were included: Mahmoudabad (population 479,433), Lyari (661,926), Manghopir (771,236), and Saeedabad (832,583) (Pakistan Bureau of Statistics, 2018). In both countries, the study was hosted by one NGO that provided psychosocial (rather than mental health) and safeguarding support to children and youth aged 4–18 years, and their families. Both NGOs were local, had direct links with statutory Social Services, and made safeguarding referrals. These services were more readily available in South Africa. Each NGO acted as gatekeeper for safeguarding in the study, facilitated recruitment, and organised the ToT programme. The project was implemented between 2022 and 2023.

### Service transformation framework

The objective of the framework was to systematically integrate child mental health awareness and promotion within the existing psychosocial support available in Global South resource-constrained settings, through co-production with local stakeholders. The framework was grounded in the theories of hierarchy of human needs (Maslow, 1943), socioecological systems (Bronfenbrenner, 1979), cross-cultural and trauma-informed care (Im & Swan, 2020), and scaled and integrated service response (World Health Organisation, 2014). Child mental health systems and capacity-building priorities in the Global South also informed its development (Vostanis et al., 2018).

**Figure 1.**
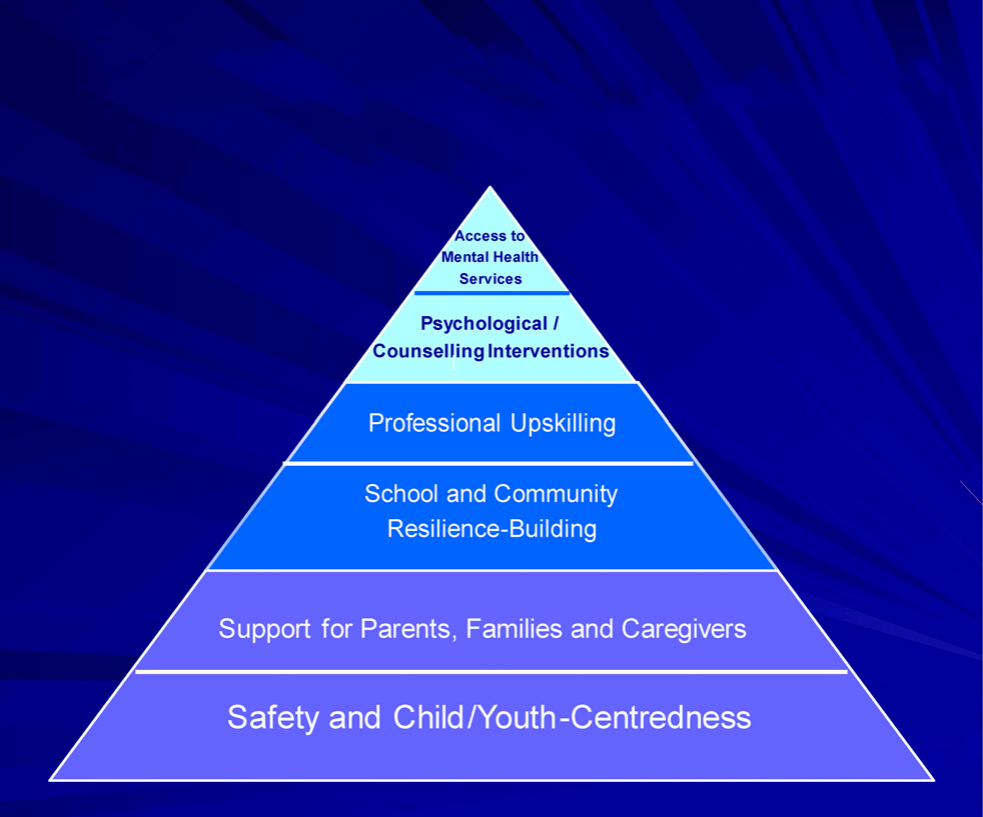
Child mental health service transformation model informing our framework.

The model informing the service transformation framework consists of five inter-linked domains along the socioecology of a child with complex mental health needs, and the support s/he would ideally require from agencies. Domains are positioned hierarchically to provide physical and emotional safety, support to parents or caregivers, resilience-building through school and community, upskilling of frontline professionals or community volunteers, and access to mental health services (Figure 1). Providers (also referred to as trainers throughout the paper) and community stakeholders subsequently co-formulate one service plan per domain, according to pre-defined criteria. The model was piloted in five Global South countries, including South Africa and Pakistan (Vostanis et al., 2019). In its pilot implementation, stakeholders prioritised mental health awareness, collaborative care, and integration within existing support systems.

Through each host NGO, practitioners operating in the target areas were invited to participate as trainers. In South Africa, four practitioners were recruited (three female, one male), and consisted of social work (1), childcare (2) and community development (1) professional background. In Pakistan, because of the wider spread of the target areas, eight trainers were recruited (six female, two male) and consisted of teacher (2), psychologist (1), psychotherapist (1), social worker (1), community worker (1), and youth activist (2).

### Train-of-Trainer (ToT) child mental health programme

The ToT programme was the same in both countries, albeit culturally adapted. Each programme included 16 two-hour sessions, provided remotely by one local and one external member of the research team of psychology and psychiatry background respectively. Intermittent reviews and regular tutorials were provided face-to-face. The programme included four modules on assessment, common child mental health problems, interventions, and systemic issues, with incorporated training on trainer skills across the modules. In the first module, one session was dedicated to the national child protection and mental health legal framework, which was revisited throughout the programme, particularly in relation to case-based discussions. The second module provided knowledge and basic skills on the key intervention frameworks in a trauma-informed context (psychodynamic, behavioural, cognitive-behavioural, narrative, attachment-focused, systemic), rather than in-depth competencies. The modules were supported by materials in the form of a manual, two e-learn modules, a range of psychoeducational resources and a practical bibliography. Booster training sessions were provided remotely fortnightly to refresh and expand on the application of newly acquired knowledge and skills throughout the implementation period.

### Implementation of service transformation model

On completion of the ToT, trainers met with community stakeholders in their target area and determined needs, resources, gaps and one priority in relation to each domain. According to the guidelines, they then co-produced a service plan for each domain that integrated child mental health into existing psychosocial support. Service plans had to be Specific, Measurable, Achievable, Realistic and Timed (SMART – Cairns et al., 2019). The service plans and interventions per country and model domain are listed in Appendix 1. The implementation of the service transformation framework by the local trainers was completed over nine months. Each intervention or training related to mental health included between 4-8 sessions. Details of each service plan and intervention are not included, as the scope of this paper is to consider implications for the overall framework, i.e., across domains and the two countries. A total *n* = 368 end-users (young people, parents, and professionals) were involved in the South Africa and *n* = 136 in the Pakistan implementation.

### Data collection

We employed the data collection method of focus groups by involving stakeholders in collaborative conversations to generate their perspectives, insights, and experiences (Adler et al., 2019). It is well-recognised that using focus groups is an especially insightful method when engaging marginalised groups who are seldom heard in policy or decision-making. This is arguably because they have an advantage of being guided conversations which encourage self-disclosure in a safe space through collaborative dialogue (Freeman, 2006).

Through purposive sampling, we invited a sub-sample of end-users from each intervention domain to attend respective focus groups following implementation (*n* = 43 in South Africa and *n* = 40 in Pakistan – Appendix 1). The topic guide explored participants’ perspectives on child mental health support, whether/how they may have shared learning within their community, and recommendations on how communities could generate and transfer mental health knowledge to enhance reach and impact. Each focus group was held at a community venue, and discussions were in *isi*Zulu and Urdu respectively, and were audio-recorded. Ethics approval was granted by the University of Leicester Research Ethics Committee in the UK. This was considered compliant with local research ethics jurisdictions after discussions with local research representatives. All participants were 16-years and over and provided written informed consent.

### Analytic approach

Data from the two studies were subject to thematic analysis, so that findings from different aspects of the process evaluation could be integrated and interpreted through a transparent coding process. We used both inductive and deductive coding approaches through a multiple coding process consistent with a codebook style of thematic analysis (Braun & Clarke, 2022). Because of our focus on synthesising the development of the transformation framework and pulling together several aspects of data collection, we deductively present the process and layers of the framework, rather than specific analytic themes derived from the data.

Thus, we do not present a traditional analysis but instead utilise data examples to illustrate the importance of a layer in the framework or demonstrate the perceived value of that element by participants. Below, we illustrate the four layers of development and showcase their importance, utilising the voices of participants who co-designed each part. The layers referred to, as the transformation framework evolved, were iteratively developed as we engaged participants in each country. These were primarily inductively built, as we generated various datasets, but were deductively informed by the wider literature and project activities.

## Results

### Layer one: Knowledge generation

As part of the development of the transformation framework (and any subsequent implementation), it is first necessary to yield knowledge about mental health from the perspectives of those central to the endeavour. Stigma of mental ill health was evident across both societies, and participants prioritised community awareness to reframe negative attitudes and beliefs, arguing that awareness was a crucial first step in mental health promotion.

“I think it [stigma] is because they don’t have enough knowledge. So, when you try something new, they will be like it’s because she thinks she is better. That is how it starts.”

South Africa, Mother with newborn 1

“Few said that it is still difficult for us to understand some concepts like stigma and managing child behaviour, because we have shed light on very minor and basic things, so things are still in their minds, it is difficult to understand, how should we do it? They think that’s right, I mean, they need more training.”

Pakistan, Trainer 7

Enhanced awareness facilitated generation of new knowledge on children’s mental health needs, factors that made them vulnerable but also protected them in their family and community environment. As emotional literacy was central to the interventions, participants reported how this had helped them approach emotionally dysregulated responses by focusing more on social contributing factors. Importantly, training was seen as valuable by the participants and as part of the solution to meeting child mental health need. Indeed, participants highlighted certain aspects of the training that improved their knowledge and the related risk factors.

“We were made aware that GBV [gender-based violence] is linked to your mental health, because if I am being violated at home, I am going to have mental health issues. I will start to think a lot. What is wrong with me that made this person do this to me? What did I do to them? Is there something wrong with me? Then I will start to suffer from depression, low self-esteem, and then it will start to affect me mentally.”

South Africa, Youth 5

“I have learned a lot to manage my emotions.”

Pakistan, Teacher 8

Such understanding was important for the earlier recognition of mental health needs. It also aided stakeholders in assessing knowledge and skills gaps in their respective groups (community, practice, services, policy) and helped them with practical strategies for coping with emotional health challenges.

“So, it hit me hard there that people actually do see, but they don’t know who it is. I hide it with a smile and jokes and everything, but when I am alone, I am this pathetic potato.”

South Africa, Youth 13

“We never thought that mental health is also an important and big component, and its effects can happen too, even though we’ve been post-violence survivor, trauma survivor, and we’re dealing with a lot of emotions, that’s how do we behave.”

Pakistan, Trainer 1

Overall, then an important aspect of the framework was knowledge generation. Participants found that raising awareness of mental health and understanding their mental health needs through improved knowledge was a central step in help-seeking, understanding, and coping.

### Layer two: Knowledge translation and transfer

An important aspect of the transformation framework involved a cascade approach of training professionals, young people and communities. For training to be engaging and transferrable, participants highlighted the importance of co-production with local communities, so that mental health was understood and connected to priorities such as poverty and lack of basic needs, unemployment, cultural and religious context, and gender inequalities.

“I think on my side, it’s the pressure, because when they talk about unemployment, there’s also the pressure that comes with it. Because you’ll see something somewhere, the pressure starts after a while. It’s all about the environment around you.”

South Africa, Peer educator 1

“We even started a series of parents training by religious scholars. It has created a difference. So, we should train more because parents, especially mothers, are not literate.”

Pakistan, Manager 3

End-users and trainers linked key aspects of the training to their lived experiences and could translate the relevance of trauma-informed and child-centred care and participation. In a parallel process, peer educators and trainers valued self-care and empowerment before supporting others.

“My role in child mental health is like a support system. First of all, we should listen to the child. We should understand everything the child says. After that, we should discuss with the child if here is need of discussing things with him. The mother should make a relationship strong with the child, which is like a friend of the mother.”

Pakistan, Mother 3

“I think I have been empowered to be able to counsel some of these youth. So, I think, yeah, the programme was good, and yeah, I was fully empowered.”

South Africa, Trainer 4

Once translated, knowledge could be cascaded by trainers to other frontline professionals and community volunteers through this structured programme. Interestingly, many parents and youth cascaded new knowledge spontaneously to their family, peer group, school, or community. This was received positively and seen to make a difference to them.

“What I share with my family is that, if you feel that you are over-stressed, you have to try to calm down and to relax, so that the stress that you have cannot lead to depression. In order to calm down and not get to a stage of depression, you have to relax. When you see that you are stressed, you have to exercise, try to do something that will help you de-stress.”

South Africa, Youth 21

“I have learnt, and I want to give these trainings to parents that they should not force the children. Try to come on a child level to understand their needs and thoughts. I hope if we train them, it will work.”

Pakistan, Teacher 6

Overall, it was recognised by different stakeholders, including the young people themselves, that absorbing knowledge alone was insufficient for learning and change. They considered how the knowledge from the training needed to be translated and contextualised to their personal situation and local context so that it could be implemented and used in positive ways. Thus, knowledge translation and transfer are central to service integration and mental health promotion.

### Layer three: Knowledge transformation and community mobilisation

Participants considered how the reach of mental health awareness and interventions could be enhanced in future. Acknowledging the extent of stigma and limited resources, the focus was on engaging and mobilising communities, in ways that moved knowledge beyond its translation to enacting change. Involving extended family and social networks, going through community and religious forums, providing safe spaces, and providing creative and social activities, were some recommendations of enabling strategies.

“I want to give an example regarding my role in the community. The day we had our training, we asked the kids to express their emotions through paint and the kids in the centre had a great time and they expressed their emotions through painting. It was a great activity session for the kids and moms too, when asked a young boy ‘what did you paint, can you tell us about your painting?’ Yes, I did. He said that when you are emotional or sad, you should express it through painting, I painted a seaside with stars, peace, and cool sand. Whenever I get sad or depressed, I paint these things. The kids understood the concept and it was clear to them that we should care about ourselves.”

Pakistan, Youth 3

“The idea is that, well, they [youth] are very creative. I remember the last one that we did, I gave them topics, types of mental health, and instead of them discussing it and presenting it, they acted it. So, they acted it in a way in how they would feel if they were living with someone with mental health, and that did show me that they are creative and that sometimes it’s okay to just let them do things how they understand them.”

South Africa, Trainer 1

Youth had a central role as peer educators in both countries. Our framework has youth at the centre, and it is critical that they are actively engaged in the process of knowledge mobilisation as agents of change, in supporting structural and service improvements in their communities. The role of educator was positively received by these youth, as they understood community needs and priorities, while their age and lived experience enabled them to relate to their peers in school and community settings. This placed them in a unique knowledge position for community mobilisation.

“Yes, we need someone close to our age to guide us and also share information, because if it is someone very stern then people won’t be open.”

South Africa, Youth 3

“I was not ready to do this conversation on child abuse with my son, but now, after listening to the girls [peer educators], they have motivated me to start educating my girls and my son, especially about good and bad touch.”

Pakistan, Mother 5

Youth-led mental health promotion, however, required ongoing training, supervision and, crucially, integration within existing services and systems. Such a role should not be confined to youth only, as parents, especially those with lived experience, expressed a wish to actively contribute.

“I want that the people who have trained us, we need more senior trainers than them. Because if we talk about emotions with someone, if someone shares their emotions with me, then if there is a question like this, I will not have an answer for it.”

Pakistan, Youth 4

“…adult supervision. When talking to your peers, you would also need an adult to come in between…for instance, I am talking about what happened that caused me to have this thing. For sure, some of your peers won’t be able to handle me, and no one will be able to handle me when I am crying.”

South Africa, Youth 14

Overall, then, our framework recognises the active role of youth and their parents. For community mobilisation and for knowledge to be truly transformed in contextualised, culturally appropriate, and meaningful ways, the community members and youth need to be in control of its direction and implementation.

### Layer four: Knowledge impact and service integration

Most end-users acknowledged the importance of interventions being complemented by systemic and structural changes for their impact to be sustained. As services were both limited and fragmented, a priority was to improve signposting and joint care pathways. The inter-agency service directory that was developed in both countries was an example of improving help-seeking. This directory referred to potential access from the target areas in each country, and showed some differences, with more statutory services included in South Africa, in contrast with more NGOs or Foundation Trusts in Pakistan. No psychiatric services were included, as these were central in both cities, rather than in the proximity of participating end-users. In particular, in South Africa, counselling or mental health support could be available through three clinical psychology centres, four primary health care services, four social relief centres, one self-harm clinic through the South Africa Depression and Anxiety Group, and one helpline (all for both adults and children). In Pakistan, the directory included two mental health services (adults and children), seven NGOs on health issues (health promotion, nutrition, child development, Down’s syndrome, autism, drug use), four NGOs on educational issues (vocational, career counselling, life skills), and one NGO on children’s rights.

“On my side, I think some parents don’t even know like adults don’t even know, what the appropriate services are.”

South Africa, Trainer 3

“So, my first thought after looking at it is that this directory will help us resolve issues of children whom we find in difficulty…I never had an idea where to send them and how they can be helped. This is a great resource.”

Pakistan, Manager 1

As children often had complex social, health and educational needs, inter-agency networks, information sharing, access to complementary professional skills, and joined up working would lead to higher quality collaborative care. Such a model would be strengthened by ongoing training in an interdisciplinary context.

“We should collaborate with local NGOs to do different programmes for community, especially for youth, because they are our future.”

Pakistan, Mother 6

“What worked well for me, it was I chose to work with other organisations, so my clients were more open, because they trusted the organisation that I came through. So, it was easy for me to engage and interact with that client.”

South Africa, Trainer 1

To accomplish this in practice, integration and impact process were needed. Integration with existing services and support has potential to increase uptake, while maximising limited resources. In resource-constrained settings, psychosocial support should be built into provision of basic needs.

“I have always worked on child protection, and I have never found it difficult to work, but the first time I connected it to mental health I faced a lot of problems. I, myself, also found it very difficult to connect and it is still like if I was working in, I would have had a problem, but when we talk about mental health, I have a lot of problems because during the training, there was problem to talk about emotions.”

Pakistan, Trainer 6

“And I think religious people that help with counselling, you know, churches or anything like that.”

South Africa Manager 3

Overall, service integration is a complex endeavour that involves multiple stakeholders, community members, families and youth. The development of our framework accounted for the importance of knowledge impact and the structural and service mechanisms that may facilitate or hinder that. Service integration needs to be driven by its key members for sustainability and structural change.

## Discussion

The goal of this paper was to present a pedagogical child mental health service transformation framework for policy makers, practitioners and researchers working in Global South resource-constrained contexts. The framework was theoretically informed, evidence-based and co-produced during a series of research projects with youth, parents, professionals and communities, and incorporates a Train-of-Trainer programme and supporting tools. We have argued that this framework is flexible and adaptable and can be used in similar contexts, by contextualising and re-focusing the training modules whereby key local people are involved in the development and delivery. However, what we have presented in this paper illustrates the value of meaningfully involving local stakeholders, the usefulness of a cascade training, and an inclusive locally driven approach to driving the agenda for change.

**Figure 2.**
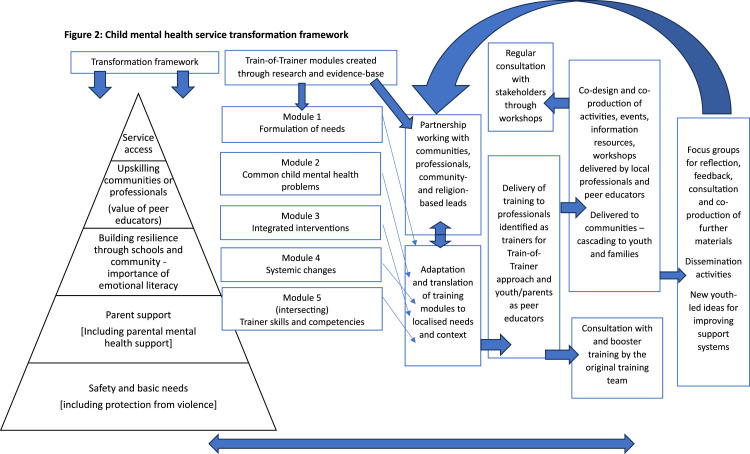
Child mental health service transformation framework.

The findings highlight the inter-linkage between the four established layers (Figure 2). Mental health awareness and stigma prevention should be ongoing, multi-dimensional (i.e., across children’s socio-ecology), and transcend other activities to enhance recognition of emerging child mental health needs and help-seeking (Hartog et al., 2020). Community- and school-based interventions should be contextualised to local realities and priorities through local partnerships (Loades et al., 2024). These should build on existing informal and structural support systems rather than uncritically import or re-create western approaches. Youth and parents with lived experience have been shown to offer a valuable first-level response role as peer educators or support workers (Rose-Clarke et al., 2019), if their roles are framed within a comprehensive service model, in which they are offered support, supervision and training. Despite the global digital divide, further development and use of digital child mental health services can complement and enhance capacity (Galagali & Brooks, 2020).

The service transformation framework was informed by the scaled and integrated care model (World Health Organisation, 2014), which is compatible with the findings and the four inter-linked layers of development and arising clinical implications. Grassroot support has significant potential, with a range of community-based organisations and paraprofessionals being available, committed and capable of providing first-level response. By holding local knowledge and trust, such agencies and groups are well positioned to promote mental health, thus engage children, youth and families with mental health services. These are, however, often fragmented, rely on short-term (partly international) funding, have too broad objectives, and lack mental health skills relevant to their psychosocial role. To maximize this potential, there is need for leadership and co-ordination, joint care pathways, and direct access to the usually overwhelmed and crisis-driven mental health services. This requires a clear model of integrated psychosocial care, such as through community- or school-based inter-agency hubs, which is embedded in intersectoral policy, workforce strategy and supporting budgets. Policy should thus tackle child mental health in the context of safeguarding, physical health, education, gender inequalities, and inclusion (Baird et al., 2021; Kruk et al., 2022).

In Global South contexts, we also argue that it is a necessity to not set rigid boundaries between applications of mental health promotion, response, and prevention (World Health Organisation, 2014). Instead, it is more helpful to develop overarching frameworks that encompass aspects of each by way of improving the mental health landscape of any country, especially those with resource constraints. Furthermore, it is more likely that local communities are tasked with managing the reality of growing child mental health needs. Notwithstanding the limitations of this body of research (such as not necessarily representative sites and samples, limited implementation period, and lack of quantitative outcome measures), the findings informed a provisional Theory of Change, which can be adapted for other resource-constrained Global South settings (Appendix 2).

In conclusion, a broad transformation framework for the integration of child mental health in practice, accounting for the mental health spectrum and providing a model founded on theory, evidence-base and contextualised principles can be a useful mechanism for local implementation of preventive and responsive psychosocial interventions for children, youth and families.

## Appendix 1

### End-users at service transformation interventions and focus group participants

**Table:**

	South Africa			Pakistan		
Service transformation framework domain	Service plan and intervention	End-users (*N* = 368)	Focus group participants (*N* = 36)	Service plan and intervention	End-users (*N* = 136)	Focus group participants (*N* = 40)
1. Safety	Promotion of safety from gender-based violence	Young women 13–19 years (*n* = 50)	9	Emotional safety awareness with youth peer educator co-facilitation	Mothers (*n* = 40)	6
2. Parent support	Mental health care	Mothers with newborns 18–30 years (*n* = 60)	6	Attachment-focused (nurturing skills) – school and community group	Vulnerable mothers (*n* = 45)	9
3. Resilience through school and community	Emotional literacy through teenage hub	Youth 14–19 (*n* = 70)	5	Youth mental health self-care	Youth 18–24 years (*n* = 25)	7
4. Upskilling communities and professionals	Peer educator led youth mental health awareness	Youth 15–24 years (*n* = 175)	Youth *n* = 7 Peer educators *n* = 5	Child mental health training	Religious teachers (*n* = 12)	9
5. Service access	Development of inter-agency service directory	Managers and senior practitioners (*n* = 13)	4	Development of inter-agency service directory	Managers and senior practitioners (*n* = 14)	9

## Appendix 2

### Provisional Theory of Change for child mental health service

#### Transformation in Global South resource-constrained settings

**Table:**
